# Preditores Clínicos e Microbiológicos de Falha Terapêutica em Infecções do Sítio Cirúrgico Esternal – Um Estudo Retrospectivo do Tipo Coorte

**DOI:** 10.36660/abc.20240464

**Published:** 2025-04-29

**Authors:** Jaqueline Fabiano Palazzo, Diego Augusto Medeiros Santos, Bruno Adler Maccagnan Pinheiro Besen, Caio Sambo, Gabriel Fialkovitz da Costa Leite, Samuel Terra Gallafrio, Danielle Menosi Gualandro, Lani Paola Bonilla Cuello, Marcus Vinicius Barbosa Santos, Tania Mara Varejão Strabelli, Pablo Maria Alberto Pomerantzeff, Fabio Biscegli Jatene, Rinaldo Siciliano

**Affiliations:** 1 Hospital das Clínicas Faculdade de Medicina Universidade de São Paulo São Paulo SP Brasil Instituto do Coração do Hospital das Clínicas da Faculdade de Medicina da Universidade de São Paulo, São Paulo, SP – Brasil

**Keywords:** Infecção da Ferida Cirúrgica, Cirurgia Torácica, Mediastinite, Falha de Tratamento

## Abstract

**Fundamentos:**

Embora as infecções do sítio cirúrgico (ISCs) esternal após cirurgias cardíacas sejam uma importante complicação, os preditores de falha terapêutica são pouco estudados.

**Objetivos:**

Avaliar preditores clínicos e microbiológicos de falha terapêutica de ISC esternal.

**Métodos:**

Pacientes que apresentaram uma ISC esternal foram retrospectivamente analisados. Foram avaliados dados relacionados a características demográficas, achados clínicos, achados laboratoriais e radiológicos iniciais e tratamento da ISC índice. O desfecho primário foi falha terapêutica, incluindo relapso da infecção (ISC esternal clínica após conclusão do tratamento) ou persistência da infecção (falha no tratamento ambulatorial com antimicrobiano). A microbiologia foi avaliada na infecção índice e no desfecho. Valores de p < 0,05 foram considerados estatisticamente significativos.

**Resultados:**

Entre os 489 pacientes incluídos, a idade média foi 58 anos, 265 (55%) eram do sexo feminino, 185 (38%) apresentaram diabetes mellitus. A prevalência da falha terapêutica foi 14% (67), ocorrendo em um tempo mediano de 174 (±41) dias após a cirurgia cardíaca índice. As causas mais comuns foram cocos gram-positivos e *Klebsiella pneumoniae.* Nenhum dos achados laboratoriais ou tomográficos torácicos apresentados durante a ISC esternal índice esteve relacionado ao desfecho. Após a análise multivariada, *Staphylococcus aureus*, Bacilos Gram-Negativos (BGN) resistentes a carbapenêmicos (GNB), fungos, diabetes mellitus e mediastinite /osteomielite foram preditores positivos de falha terapêutica.

**Conclusões:**

BGN resistentes a carbapenêmicos, fungos e S. aureus emergentes foram associados a um maior risco de falha terapêutica na ISC esternal. Além disso, DM e infecções profundas de lesões esternais foram fatores contribuintes. Suas implicações clínicas e o papel exato dos microrganismos multirresistentes requerem mais estudos.

## Graphical abstract

**Figura Central:**
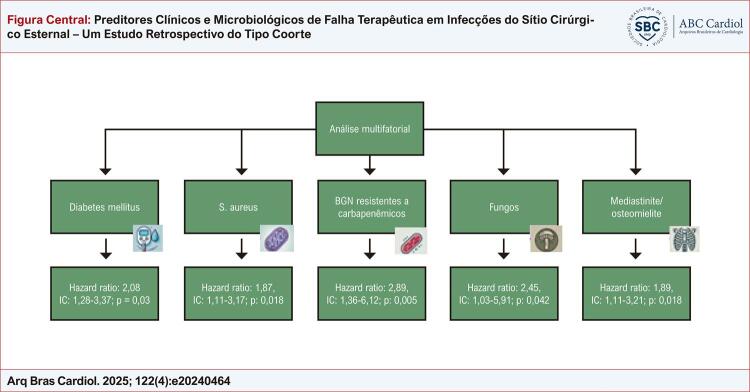
Preditores Clínicos e Microbiológicos de Falha Terapêutica em Infecções do Sítio Cirúrgico Esternal – Um Estudo Retrospectivo do Tipo Coorte

Análise multivariada de fatores associados com falha terapêutica nas infecções de sítio cirúrgico esternal.

## Introdução

A infecção em sítio cirúrgico (ISC) é uma complicação pós-operatória importante, representando a terceira maior causa de infecção associada à assistência à saúde.^1^ Embora mais de 50% das infecções das feridas operatórias sejam preveníveis,^2^ elas representam a principal causa de internação prolongada (com períodos de internação mais de duas vezes mais longos), e uma das principais causas de reinternação.^3-6^ Assim, a ISC constitui uma carga financeira com impacto negativo sobre a qualidade de vida e todos os sistemas de saúde, aumentando os custos em até 240%.^7-10^

Entre as ISCs, a infecção da ferida esternal é um evento particularmente preocupante devido à potencial rápida progressão para mediastinite.^11-13^ Apesar de sua baixa incidência, variando entre 0,5-5,0%, a mediastinite pós-cirúrgica tem uma taxa de mortalidade alta, entre 15 e 47%, em contraste à mortalidade geral de 2-4% por outras complicações cardiológicas.^14-18^ Ainda, o tratamento da mediastinite pós-cirúrgica é complexo, e geralmente inclui desbridamento, uso precoce e adequado de antibióticos, e fechamento com retalho muscular.^1 9-23^

A ISC recorrente é comum, com estudos prévios relatando uma taxa de ocorrência entre 2% e 60%.^23-26^ Como esses estudos focam principalmente em comparar técnicas cirúrgicas e o manejo de feridas esternais, pode-se encontrar uma ampla gama de definições de reinfecção/recorrência de infecção, o que dificulta a estimativa de sua real prevalência. Apesar de sua importância clínica, há escassez de estudos com foco em fatores de risco para recorrência de infecção. Diante disso, o objetivo do presente estudo é avaliar preditores clínicos e microbiológicos para falha terapêutica na infecção da ferida esternal.

## Métodos

Este é um estudo retrospectivo conduzido em um centro de cardiologia quaternário. Trata-se de um hospital com 70% dos leitos destinados a pacientes cirúrgicos, em que são realizadas 3800-4000 cirurgias cardíacas anualmente. O estudo foi aprovado pelo comitê de ética local (número 31593814.8.0000.0068); o consentimento do paciente não foi obtido pela natureza do estudo.

Entre janeiro de 2016 e dezembro de 2019, foram avaliados pacientes com diagnóstico de ISC após serem submetidos à cirurgia cardiotorácica. Pacientes com idade inferior a 15 anos ou que foram a óbito durante a mesma internação hospitalar foram excluídos. ISC esternal foi definida de acordo com *Centers for Disease Control and Prevention* (CDC) / *National Healthcare Safety Network* (NHSN).^27^Todos os pacientes com evidência clínica de ISC esternal foram avaliados por uma equipe multidisciplinar composta por cirurgião plástico, cirurgião cardíaco e especialista em doenças infecciosas. A equipe de controle de infecções realiza diariamente uma vigilância ativa em unidades de internação e visitas médicas após a alta para a avaliação de ISC e orientação na fase final da terapia. Protocolos institucionais recomendaram regime empírico de antibiótico, geralmente com vancomicina e quinolona, de acordo com recomendações internacionais.^19^

A duração do tratamento foi de 30 dias ou mais em pacientes submetidos a desbridamento cirúrgico. Todas as mudanças nos procedimentos com antimicrobianos foram feitas de acordo com as orientações do infectologista.

O Dia 0 foi o primeiro dia de sintomas clínicos, correspondendo ao dia de inclusão no estudo. Os pacientes foram acompanhados em consultas ambulatoriais regulares até julho de 2021.

Os pacientes encaminhados a outras instituições foram contatados por telefone para verificar seus estados de saúde e convidados a comparecerem no hospital se necessário. Mediastinite/osteomielite pós-esternotomia foi definida de acordo com os CDC/NHSN,^28^ caracterizando infecções de lesões esternais profundas. Um dos seguintes critérios era necessário: (1) cultura bacteriana positiva de tecido mediastinal/ósseo; (2) evidência clínica de mediastinite/osteomielite durante a cirurgia; ou (3) uma das seguintes condições clínicas – dor torácica, instabilidade do esterno, febre (38^o^C) e secreção purulenta do mediastino ou achados radiológicos sugestivos de mediastinite/osteomielite. ISC índice foi o primeiro episódio infeccioso na esternotomia mediana. A cirurgia cardíaca índice foi a última abordagem cardíaca. Quanto ao tratamento da ISC índice, foram avaliados desbridamento cirúrgico inicial e tempo para terapia efetiva com antibiótico, definida de acordo cm o perfil de sensibilidade da bactéria. O período de incubação foi definido como o período entre a cirurgia cardíaca índice e desbridamento inicial ou o dia do início de um antibiótico empírico para casos tratados não cirurgicamente.

Os dados foram coletados do registro hospitalar e os pacientes avaliados por sexo, idade e presença de Diabetes Mellitus (DM). Dados laboratoriais foram coletados em até três dias após o Dia0, e dados tomográficos considerados válidos se obtidos em até sete dias do Dia0. Os pacientes foram classificados de acordo com o tipo de cirurgia e se houvesse histórico de esternotomia mediana.

Somente culturas obtidas por técnica estéril do dreno da ferida ou da biópsia tecidual foram aceitas para análise microbiológica. Todos os isolados bacterianos foram identificados por espectrometria de massa e a sensibilidade foi testada usando o sistema MS Vitek 2 system (BioMe ´rieux). Os perfis de resistência foram definidos de acordo com o M100-S25 do *Clinical and Laboratory Standards Institute* (CLSI). A resistência aos carbapenêmicos foi considerada se fosse identificada resistência a imipenem, meropenem ou ertapenem. Outro padrão de resistência considerado foi o de *Staphylococcus aureus* resistente à oxacilina. Estafilococos coagulase-negativos (ECN) e contaminantes de pele foram considerados válidos microbiologicamente somente se estivessem presentes duas ou mais amostras obtidas em ocasiões separadas. Infecção de corrente sanguínea (ICS) foi definida de acordo com as definições do CDC/NHSN para ICS secundária.^27^

Nosso desfecho primário foi falha terapêutica da ISC esternal índice, definida como relapso da infecção (recorrência do quadro clínico de ISC no esterno, com reinício de antibióticos após a resolução clínica), ou persistência da infecção (tratamento antimicrobiano ambulatorial e readmissão hospitalar). Os pacientes que apresentaram diferentes culturas de microrganismos durante o desfecho foram considerados como reinfecção e não como falha terapêutica.

### Análise estatística

Os dados categóricos foram resumidos como número e porcentagem. A distribuição dos dados contínuos foi avaliada por histogramas e pelo teste de Shapiro-Wilk. Médias e desvios padrões dos dados com distribuição normal e medianas e Intervalos Interquartis (IIQs) dos dados assimétricos foram descritos. O teste t de *Student* para dados assimétricos foi usado para comparar variáveis com distribuição normal, e o teste de Wicoxon para variáveis contínuas sem distribuição normal. O teste exato de Fisher foi usado par variáveis categóricas. Um conjunto de variáveis preditoras que poderiam estar associadas com o desfecho foi pré-definido. Realizamos um rastreamento univariado com um ponto de corte para o p-valor de 0,1. As variáveis preditoras foram incluídas em um modelo de regressão multivariada de Cox. Em seguida, realizamos um procedimento de seleção *backwards*, escolhendo o melhor modelo de ajuste de acordo com os Critérios de Informação de Akaike (AIC) e Bayesiano (AIB). Avaliamos o pressuposto de riscos proporcionais usando o gráfico log-log e resíduos de Schoenfeld. A curva de incidência cumulativa foi construída pelo método Nelson-Aalen. Os pacientes foram incluídos somente uma vez, no Dia0, e o tempo final definido como morte por qualquer causa ou primeira ocorrência do desfecho. A análise estatística foi realizada usando o programa Stata SE 16.0; valores p < 0,05 foram considerados estatisticamente significativos.

## Resultados

**Figura 1:**
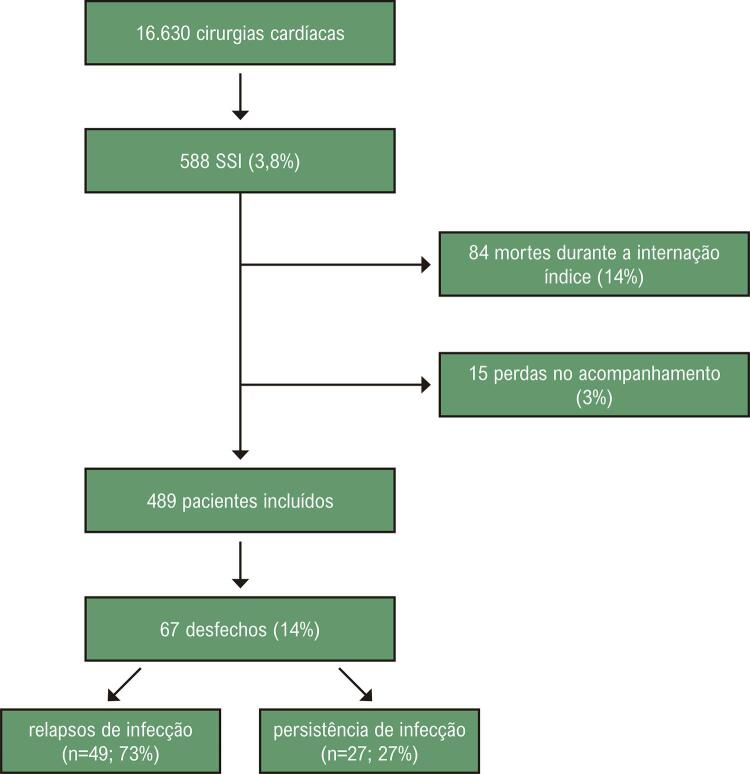
– Diagrama do estudo e critérios de exclusão.

**Figura 2:**
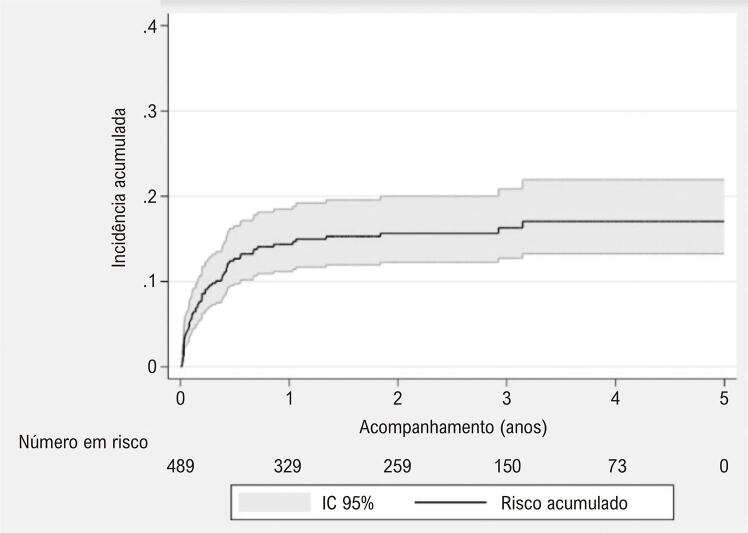
– Curva de Nelson-Aalen mostrando a incidência acumulada.

Durante o período do estudo, 16 330 cirurgias cardíacas foram realizadas e 588 episódios de ISC esternal (3,6%) foram identificados. Entre esses, 14% (84/588) foram excluídos por morte durante a internação índice e 3% (15/588) por perda de seguimento (Figura 1). Dos 489 restantes, o desfecho primário ocorreu em 14% (67/489) em uma mediana de 174 (IIQ ± 41,2) dias após a cirurgia cardíaca índice. Entre eles (49/67) foram considerados relapsos de infecção, e 27% (18/67) persistência da infecção, com a última ocorrendo em 15 (IIQ ± 36) dias. O período médio de acompanhamento foi de dois anos (0,002- 489). Dados clínicos, demográficos e terapêuticos da ISC índice são apresentados na Tabela 1 e a incidência cumulativa na Figura 2.

**Tabela 1:** – Características da população do estudo e análise univariada

Características basais / Variáveis	Todos os pacientes n=489 (%)	Falha terapêutica n=67 (13,7%)	Sem falha terapêutica n=422 (86,2%)	IC95%	Valor p
**Idade média, anos ≥50 anos**	58,3 (±15) 377 (77,1)	60,8 (±14) 57 (85,0)	57,9 (±15) 10 (14,9)	0,93-3,57	0,14 0,059
**Sexo feminino**	265 (54,2)	40 (59,7)	225 (53,3)		0,36
**Diabetes mellitus**	185 (37,8)	36 (53,7)	149 (35,3)	1,25-3,28	0,003
**Cirurgia cardíaca índice**					
*Bypass* da artéria coronária	229 (54,0)	40 (69,0)	189 (51,6)	1,11-2,97	0,014
Esternotomia mediana prévia	54 (11,0)	10 (14,9)	44 (10,4)		0,29
**Apresentação clínica e tratamento da ISC índice**					
Período de incubação ≥28 dias	94 (19,2)	13 (19,4)	81 (19,2)		1,00
Dreno purulento da ferida	427 (88,4)	63 (96,9)	364 (87,1)	1,08-18,1	0,007
Deiscência ≥ 3cm	52 (10,6)	7 (10,4)	45 (10,7)		1,00
Febre	118 (24,5)	21 (31,7)	97 (23,4)		0,17
Infecção de corrente sanguínea secundária	89 (19,8)	18 (26,9)	71 (16,)	0,99-2,93	0,06
Mediastinite/osteomielite	88 (18,0)	22 (32,8)	66 (15,6)	1,41-3,93	0,002
Demora em iniciar antibioticoterapia efetiva ≥ 3 dias^b^	93 (19,0)	19 (28,4)	64 (17,5)	1,01-2,94	0,05
Cirurgia de desbridamento	259 (52,9)	42 (62)	217 (51,4)		0,12
**Achados radiológicos**					
Desalinhamento anormal	9 (1,9)	3 (4,5)	6 (1,4)		0,11
Diástase/deiscência esternal	34 (7,0)	5 (7,5)	29 (6,9)		0,8
Esclerose/erosão esternal	9 (1,8)	0	9 (2,1)		0,62
Reabsorção do esterno	9 (1,8)	1 (1,5)	8 (1,9)		1,00
Pré-esternal^c^	131 (26,8)	20 (29,9)	111 (26,4)		0,55
Pós-esternal^c^	205 (42)	29 (43,3)	176 (41,8)		0,89
**Etiologia da ISC índice**					
Cultura positiva	337 (68,9)	52 (77,6)	285 (67,5)		0,12
S.aureus SARM	108 (22,1) 18 (3,7)	23 (34,3) 5 (7,5)	85 (20,1) 13 (3,1)	1,14-3,15	0,016 0,085
CoNs	148 (30,3)	16 (23,9)	132 (31,3)		0,25
Streptococcus	11 (2,2)	1 (1,5%)	10 (2,4)		1,00
*BGN BGN resistentes a* carbapenêmicos	127 (26) 25 (5,1)	26 (38,8) 8 (11,9)	101 (23,9) 17 (4,0)	1,30-5,72	0,016 0,018
Fungos	23 (4,7)	6 (9,0)	17 (4,0)	0,94-5,06	0,09
Enterococcus species	27 (5,5)	4 (6,0)	23 (5,5)		0,78
Contaminantes na pele	15 (3,1)	3 (4,5)	12 (2,8)		0,44
**Valores laboratoriais no Dia 0**					
Creatinina (mg/dL)		1,34 (±1,1)	1,32 (±1,32)		0,94
Proteína C-reativa (mg/L)		123,3 (±90,0)	119,6 (±96,3)		0,79
Leucócitos (mg/L)		11,022 (±4782)	10,826 (±4845)		0,77
Plaquetas (mg/L)		387.983 (±131)	300.977 (±136)		0,5

IC: intervalo de confiança; OR: odds ratio; S. aureus: Staphylococcus aureus; SARM: Staphylococcus aureus resistente à meticilina; ECN: estafilococos coagulase-negativos; BGN: bacilos gram-negativos; ISC: infecção de sítio cirúrgico; Contaminantes da pele: Cutibacterium acnes, Corynebacterium spp., Micrococcus luteus, Propionibacterium sp.; a: infecção de sítio cirúrgico; b: ajuste de antibióticos devido à resistência bacteriana; c: coleção de fluidos e edema de tecidos moles.

Quanto à ISC eternal índice, o período de incubação foi de 16 dias (± 10) e o tempo para a terapia efetiva com antibióticos foi de seis dias (± 2,8 dias). Um total de 259 de 489 pacientes (53%) foram submetidos a um desbridamento inicial na ISC índice. O tempo entre D0 e o primeiro desbridamento foi de dois dias (±1,5), e o tempo para o fechamento externo foi 14 dias (±0,7). No subgrupo de pacientes com ISC esternal índice classificado como mediastinite/osteomielite, 93% (82/88) foram desbridamento na ICS esternal índice, com participação da equipe de cirurgia plástica em 92% (76/82) dos casos em todos, realizou-se terapia de fechamento assistido por vácuo. Entre os pacientes com falha terapêutica da ICS esternal índice, 62% (42/67) haviam se submetido a desbridamento inicial para tratamento da ICS índice. Entre os 38% dos pacientes (28/67) que receberam uma abordagem conservadora inicialmente, 42% (12/28) foram submetidos a desbridamento tardio devido a essa falha terapêutica prévia. Osteomielite ocorreu em 31% (21/67) de todos os desfechos e em 95% (21/22) das falhas terapêuticas no grupo mediastinite/osteomielite.

Culturas de 84% (413/489) das ISCs esternais índices foram obtidas; 84,5% (347/413) delas eram positivas, entre as quais 6,3% (21/347) foram consideradas contaminação e foram excluídas. Os microorganismos mais comumente encontrados foram S. aureus (22%), ECN (30 %) e bacilos gram-negativos (BGN) (26%). Das BGN, *Enterobacteriaceae* foram 85%, com *Klebsiella pneumoniae* correspondendo a 41% e bactérias não fermentadoras 21%, com *Pseudomonas aeruginosa* representando 52% delas. Infecções polimicrobianas (≥ 2 etiologias) ocorreram em 25% (83/347).

As infecções por BGN foram as principais causas de falha terapêutica, responsáveis por 58% (39/67) dos desfechos. Entre as 39 ocorrências de falha terapêutica, 29 foram classificadas como relapso de infecção e 10 como persistência de infecção. S. aureus foi responsável por 59% (23/39) das falhas terapêuticas, 70% (16/23) foram submetidos a desbridamento para tratamento da ISC índice. Infecção por BGN ocorreu em 39% (26/67) das falhas terapêuticas – 16 foram casos de relapso de infecção e 10 de persistência de infecção. *Enterobacteriaceae* foram responsáveis por 76% (20/26) dos desfechos, sendo 70% (14/20) por *Klebsiella pneumoniae.* Entre os oito desfechos causados por *Enterobacteriaceae* resistentes a carbapenêmicos, 75% (6/8) foram causados por *Klebsiella pneumoniae* e 25% (2/8) por *Pseudomonas aeruginosa*. Desbridamento da ISC índice foi realizado em 100% (8/8) das falhas terapêuticas em BGN resistentes a carbapenêmicos. Vinte e três infecções foram causadas por fungos e mais de 90% (21/23) foram causadas por *Candida spp*., com uma prevalência de 66% (14/21) de *C. albicans*. Somente um caso foi devido à infecção por *Aspergillus fumigatus* e um devido a *Trichosporon asahii.* Todas as oito falhas terapêuticas foram por *C. albicans*, sendo que 50% (4/8) foram consideradas persistência de infecção. O desbridamento cirúrgico inicial havia sido realizado em 83% (5/6). As variáveis que continuaram estatisticamente significativas são apresentadas na Tabela 2 e na Figura Central.

**Tabela 2:** – Modelo multivariado de Cox dos fatores associados com o tempo para falha terapêutica

Variáveis	Hazard-ratio	Intervalo de confiança de 95%	Valor p
Diabetes mellitus	2,08	1,28- 3,37	0,003
S. aureus	1,87	1,11-3,17	0,018
Bacilos gram-negativos resistentes a carbapenêmicos	2,89	1,36-6,12	0,005
Fungos	2,47	1,03- 5,91	0,042
Mediastinite/osteomielite	1,89	1,11-3,21	0,018

## Discussão

O presente estudo sugere um importante papel da etiologia de ISC esternal na falha terapêutica, principalmente se BGN resistentes a carbapenêmicos estejam envolvidos. Além disso, infecção profunda de ferida esternal e DM também pioraram o prognóstico da ferida.

A taxa global de falha terapêutica foi de 14%; escolhermos adotar uma definição abrangente, com combinação de desfechos, enfatizando a dificuldade em se tratar ISC esternal. Recorrências de ISC foram discutidas em estudos prévios comparando técnicas cirúrgicas para o manejo de feridas esternais,^23-26^ geralmente sem uma definição clara. Um estudo retrospectivo do tipo coorte envolvendo 43 casos de mediastinite pós-cirúrgica^25^ apresentou um total de 31% de reinfecções, com um período mediano de acompanhamento de quatro anos. Neste estudo, reinfecção foi definida como uma infecção de ferida esternal profunda após pelo menos uma tentativa de tratamento adequado ou nova intervenção cirúrgica após a alta hospitalar. Outra revisão retrospectiva de 118 mediastinite pós-esternotomia^23^ relatou uma taxa de reinfecção de 9%, sem especificar a definição de desfecho. Em outro estudo,^24^ 101 pacientes com mediastinite pós-esternotomia, confirmada por cultura, foram analisados retrospectivamente, com uma taxa de recorrência de fístula esternal de 6%. Outro estudo do tipo coorte com 92 pacientes^26^ relatou uma taxa de reinfecção de 9,7%. Nesse estudo,^26^ recorrência de infecção foi definida como secreção purulenta, cultura de sangue positiva na presença de deiscência esternal e sinais sistêmicos de sepse. Um estudo^20^ com 267 casos de infecções de feridas esternais profundas também relataram uma taxa de recorrência de infecção de 9,7%, sem uma definição específica de recorrência de infecção. Além disso, em nossa população, a maior proporção de relapsos de infecção com uma ocorrência mais tardia (cerca de seis meses) é compatível com a alta taxa de osteomielite envolvida, e sugere uma possível invasão óssea subclínica/não suspeita em relação à primeira ISC esternal.

DM é um fator de risco reconhecido para ICS esternal, por dificultar a cicatrização óssea e de feridas, influenciando negativamente a vascularização e a função imune.^12-16^ O estudo atual reforça o papel do DM na piora do prognóstico da infecção, evidenciando-o como um fator de risco para falha terapêutica.

O desbridamento cirúrgico é atualmente considerado a terapia principal para mediastinite pós-operatória, com o fechamento assistido por vácuo mostrando resultados favoráveis quanto à diminuição da recorrência de infecção.^23,25,26^ Um estudo coorte^23^ com 118 pacientes relatou uma redução significativa na taxa de reinfecção entre os pacientes tratados com fechamento assistido por vácuo em comparação aos tratados com terapia convencional, de 18% para 2,9%.^23^ Uma redução na taxa de reinfecção de 60% para 10% com terapia assistida por vácuo foi observada em um estudo coorte com 43 pacientes.^25^ Outro estudo relatou uma menor taxa de mortalidade em 90 dias quando o fechamento assistido por vácuo foi usado em comparação a técnicas convencionais entre 101 pacientes,^24^ mas sem diferença significativa na recorrência de fístula esternal. No presente caso, mais de 90% dos casos de mediastinite/osteomielite foram tratados com desbridamento.

Em relação às análises microbiológicas, *S. aureus* foi o segundo patógeno mais prevalente envolvido na ISC esternal, mas a causa mais prevalente de falha terapêutica e um fator de risco independente dessa complicação. O NHSN^29^ descreveu o *S. aureus* como o patógeno mais comum na ISC, principalmente para cirurgias ortopédicas, obstétricas/ginecológicas, e cardíacas, com uma incidência de 27% nessas últimas. Um estudo^30^ coorte relatando 126 casos de mediastinite pós-esternotomia encontrou uma maior proporção de ECN (46%) e *S. aureus* como a segunda causa principal (26%). Além disso, 109 infecções de lesão esternal também apresentaram uma prevalência um pouco mais alta (36,7%) em comparação a S. aureus (30%) em outro estudo.^31^Outro estudo do tipo coorte de 291 infecções de ferida esternal^12^ também relatou que o *S. aureus* foi o segundo patógeno mais comum (16,5%), causando 80% dos casos de mediastinite pós-cirúrgica. Uma revisão sistemática de mais de 3500 osteomielites^32^ encontrou que *S. aureus* esteve relacionado com um risco mais alto de falha terapêutica em pacientes com osteomielite vertebral. A literatura também já chamou a atenção para mediastinite causada por *Staphylococcus aureus* resistente à meticilina (SARM),^33,34^ porém, em nosso estudo, o papel do SARM na falha terapêutica não foi avaliado devido à sua baixa prevalência.

Mediastinite pós-cirúrgica causada por fungo tem uma incidência que varia entre 1,6% e 7,5% nos poucos artigos existentes.^35,36^ Uma série de relatos de caso de 11 pacientes com infecções de feridas esternais profundas causadas por *C. albicans*^36^ mostrou uma alta prevalência de osteomielite: seis pacientes apresentaram osteomielite esternal, um apresentou osteomielite e mediastinite, e quatro apresentaram infecções de feridas profundas que provavelmente envolviam osso. Ainda, três pacientes sofreram relapso de infecção após seis meses de terapia antifúngica efetiva. Uma revisão de 76 casos de infecções de ferida esternal causada por *Candida spp*.^37^ encontrou uma taxa de 33% de relapso de infecção, definido como reintervenção. No presente estudo, observou-se uma prevalência de 4,7% de *Candida spp*. Na ISC esternal. Apesar de sua baixa incidência, a infecção por *Candida spp*. Parece ser um fator de risco independente para falha terapêutica.

BGNs resistentes aos carbapenêmicos emergentes são geralmente relatadas em infecções associadas à saúde,^29^ principalmente em infecções associadas ao dispositivo, com poucos estudos avaliando sua participação na ISC.^38,39^ Um estudo prospectivo do tipo coorte com 50 pacientes^38^ avaliou infecção por *Enterobacteriaceae* resistente a carbapenêmicos em ISC abdominal e encontrou uma taxa de mortalidade mais alta associada a tumor sólido/metástase, choque séptico e transfusão de sangue. Um estudo nacional multicêntrico do tipo coorte, conduzido na Arábia Saudita,^39^ mostrou uma prevalência de 73% de ISC por BGN após *bypass* coronário, com uma incidência de até 10,8% de resistência antimicrobiana (incluindo resistência a cefalosporina, carbapenêmicos e multidrogas). Outro estudo do tipo coorte com 33 pacientes mostrou uma taxa de mortalidade de 33% associada com mediastinite pós-cirúrgica causada por BGN resistente a carbapenêmicos.^40^ Em um estudo^41^ do tipo coorte com 142 pacientes com infecções de pele e tecidos moles causadas por *Enterobacteriaceae* resistente a carbapenêmicos, 26 (24,5%) eram ISC, sendo o segundo tipo mais prevalente de lesão. Além disso, o estudo mostrou uma taxa de mortalidade hospitalar de 15,5%, e que aproximadamente metade dos sobreviventes receberam alta para outro local para receberem suporte clínico mais intensivo. Nosso estudo sugere que, apesar de sua prevalência mais baixa, a infecção por BGN resistentes a carbapenêmicos é um fator de risco para falha terapêutica na ISC esternal, uma vez que foi detectada entre pacientes com alta prevalência de desbridamento cirúrgico, fechamento assistido por vácuo e terapia com antibióticos prolongada. Esse resultado é plausível, considerando as opções terapêuticas restritas e as condições clínicas mais complexas associadas a essa etiologia.

### Limitações

Dado o seu delineamento retrospectivo, os autores não conseguiram evitar totalmente as variáveis de confusão, tirar conclusões quanto à causalidade, e avaliar o status funcional ou o progresso clínico dos pacientes. Além disso, por ser um estudo unicêntrico do tipo coorte, não foi possível alcançar validação externa.

## Conclusão

A presença de BGN resistentes a carbapenêmicos, fungos e *S. aureus* foi associada a um risco mais alto de falha terapêutica da ISC esternal. Além disso, DM e infecções profundas de lesões esternais foram fatores contribuintes. Suas implicações clínicas e o papel exato dos microrganismos multirresistentes requerem mais estudos.
