# Supplementary material for: Preditores Clínicos e Microbiológicos de Falha Terapêutica em Infecções do Sítio Cirúrgico Esternal – Um Estudo Retrospectivo do Tipo Coorte
Source: Arq Bras Cardiol. 2025 Apr 29;122(4):e20240464. [Article in Portuguese] doi: 10.36660/abc.20240464 (PMC12107816; doi:10.36660/abc.20240464)
Supplement: Translation

# Translation: Clinical and Microbiology Predictors for Therapeutic Failure in Sternal Surgical Site Infections - A Retrospective Cohort Study

**DOI:** 10.36660/abc.20240464i

**Authors:** Jaqueline Fabiano Palazzo, Diego Augusto Medeiros Santos, Bruno Adler Maccagnan Pinheiro Besen, Caio Sambo, Gabriel Fialkovitz da Costa Leite, Samuel Terra Gallafrio, Danielle Menosi Gualandro, Lani Paola Bonilla Cuello, Marcus Vinicius Barbosa Santos, Tania Mara Varejão Strabelli, Pablo Maria Alberto Pomerantzeff, Fabio Biscegli Jatene, Rinaldo Siciliano

**Affiliations:** 1 Brazil Instituto do Coração do Hospital das Clínicas da Faculdade de Medicina da Universidade de São Paulo, São Paulo, SP – Brazil

**Keywords:** Surgical Wound Infection, Thoracic Surgery, Mediastinitis, Treatment Failure

## Abstract

**Background:**

Although sternal surgical site infections (SSI) are an important complication after cardiac surgeries, predictors of treatment failure are poorly studied.

**Objectives:**

The aim of this study is to assess the clinical and microbiology predictors of a sternal SSI therapeutic failure.

**Methods:**

Patients who presented a sternal SSI were retrospectively analyzed. Data regarding demographic characteristics, clinical findings, initial laboratory and radiologic findings and treatment of index sternal SSI were evaluated. Primary outcome was treatment failure, comprising infection relapse (clinical sternal SSI after complete treatment) or infection persistence (outpatient antimicrobial treatment failure). The microbiology was assessed at the index infection and in the outcome. P-values < 0.05 were considered statistically significant.

**Results:**

Among 489 included patients, mean age was 58 years, 265 (55%) were female, 185 (38%) had diabetes mellitus. The overall prevalence of therapeutic failure was 14% (67), occurring in a median of 174 days (±41) after index cardiac surgery. Most frequent etiologies were cocci Gram-positive and Klebsiella pneumoniae. None of laboratory or thoracic tomographic findings presented during the index sternal SSI was related to outcome. After multivariate analysis, Staphylococcus aureus, carbapenem-resistant Gram-negative bacilli (GNB), fungi, diabetes mellitus and presence of mediastinitis/osteomyelitis were positive predictors of therapeutic failure.

**Conclusions:**

Emerging carbapenem-resistant GNB, fungi and S. aureus were etiologies associated with higher risk of therapeutic failure in sternal SSI. DM and deep sternal wound infections were also contributing factors. Its clinical implications and the exact role of multi-resistant microorganism itself are subject for more studies.

## Introduction

Surgical site infection (SSI) is a major postoperative complication, representing the third cause of health care-associated infection.^1^ Even though more than 50% of wound infections are preventable,^2^ they represent the main cause of prolonged hospital stay, leading to a hospitalization period more than twice longer, and one of the main causes of hospital readmission.^3-6^ Therefore, SSI constitutes a financial burden and negatively impacts quality-of-life and healthcare systems, increasing costs by up to 240%.^7-10^

Among SSIs, sternal wound infection is an event of special concern due to its potential rapid progression for mediastinitis. Although its low incidence, ranging from 0.5-5%,^11-13^ a postsurgical mediastinitis has an associated mortality rate as high as 15-47%, in contrast to 2-4% overall mortality due to other postoperative cardiologic complications.^14-18^ Moreover, the treatment of postsurgical mediastinitis is complex and generally includes debridement, earlier and adequate intravenous antibiotics and flap closure of mediastinum.^19-23^

Recurrent sternal SSI is common, with an occurrence rate ranging from 2% to 60%.^23-26^ As these studies mainly focus on comparing surgical techniques and management of sternal wounds, a wide range definitions of re-infection / infection recurrence can be found, which makes it difficult to assess its real prevalence. Despite its clinical importance, there is a lack of studies focused on risk factors for infection recurrence. Considering this, the aim of the current study is to assess clinical and microbiology predictors for therapeutic failure in sternal SSI.

## Methods

This is a retrospective cohort study conducted in a quaternary cardiology center. This is a hospital with 70% of its beds for surgical patients, in which 3800-4000 cardiac surgeries are performed yearly. Ethical approval was given by the local committee (registration number 31593814.8.0000.0068). Patients’ consent was not taken due to the retrospective nature of the analysis.

From January 2016 to December 2019, patients diagnosed with SSIs after cardiothoracic surgery were evaluated. Patients aged under 15 years or who died during the same hospitalization were excluded. Sternal SSI was defined in accordance with the Centers for Disease Control and Prevention (CDC) / National Healthcare Safety Network (NHSN).^27^ All patients with clinical evidence of sternal SSI were evaluated by a multidisciplinary team consisting of plastic surgeon, cardiac surgeon and infectious diseases specialist. The infection control team performs daily active surveillance in inpatient units and post-discharge medical visits for SSI evaluation to guide the end of therapy. Institutional protocols recommended empiric antibiotic regimen usually with vancomycin plus quinolone, according to international recommendations.^19^

Treatment duration was at least 30 days in patients undergoing surgical debridement^.^ All changes in antimicrobial procedures were made according to the infectologist’s instructions.

Day 0 was the first day of clinical symptom, corresponding to study inclusion. Patients were followed up in outpatient clinics with regular visits until July 2021.

Patients who were referred to other institutions were contacted by telephone to check their health status and invited to hospital if needed. Post-sternotomy mediastinitis/osteomyelitis were defined in accordance with CDC/NHSN,^28^ characterizing deep sternal wound infections. It was required at least one of the following criteria: (1) positive bacterial culture from mediastinal/bone tissues; (2) clinical evidence of mediastinitis/osteomyelitis during surgery; or (3) one of the following clinical conditions – chest pain, sternal instability, fever (38°C) and purulent discharge from the mediastinum or radiologic findings suggestive of mediastinitis/osteomyelitis. Index SSI was the first infectious episode in median sternotomy. Index cardiac surgery was the last cardiac approach. Regarding index SSI treatment, initial surgical debridement and time to effective antibiotic therapy were assessed, which was established in accordance with bacterial sensitivity profile. Incubation period was defined as the period between index cardiac surgery and initial debridement or day of initiation of an empirical antibiotic for non-surgical treated cases.

Data were collected from the hospital registry and patients were evaluated by gender, age and presence of diabetes mellitus (DM). Laboratorial data were collected within three days of Day0 and tomographic data were considered valid if collected within seven days of Day0. Patients were classified according to the type of index surgery and if there was any previous median sternotomy.

Only cultures obtained by sterile technique from wound drain or tissue biopsy were accepted for microbiology analyses. All bacterial isolates were identified by mass spectrometry and had the sensitivity tested using the MS Vitek 2 system (BioMe ´rieux). Resistance profiles were defined according to M100-S25 of the Clinical and Laboratory Standards Institute (CLSI). Carbapenem resistance was considered if any carbapenem resistance (imipenem, meropenem, or ertapenem) was identified. Another resistant pattern of concern was oxacillin-resistant *Staphylococcus aureus*. Coagulase-negative *Staphylococcus spp.* (CoNS) and skin contaminants were microbiologically valid only if present ≥ 2 samples drawn on separate occasions. Bloodstream infection (BSI) was defined in accordance with CDC/NHSN definitions of secondary BSI.^27^

Our primary outcome was therapeutic failure of index sternal SSI, defined as: infection relapse (resumption of the clinical picture of sternal SSI, restarting antibiotic after clinical resolution) or infection persistence (outpatient antimicrobial treatment failure and hospital readmission). Patients who had a different microorganism cultured during the outcome were considered as reinfection and not taken as therapeutic failure.

### Statistical analysis

Categorical data were summarized as number and percentage. Data distribution of continuous data was assessed through histograms and the Shapiro-Wilk test. The mean and standard deviations were reported for normally distributed data, and median and interquartile ranges (IQRs) for skewed data. The unpaired Student’s t-test was used to compare normally distributed variables and the Wilcoxon rank-sum test for non-normally distributed continuous variables. The Fisher exact test was used for categorical variables. A priori a set of predictor variables that could be associated with the outcome was defined. We performed a univariate screening with a *p*-value cutoff of < 0.1. Those predictor variables were included in a multivariable Cox regression model. Then, a backwards purposeful selection procedure, choosing the best fitted model with Akaike and Bayesian information criteria (AIC/BIC) was used. We evaluated the proportional hazards assumption with log-log plots and Schoenfeld residuals. The cumulative incidence curve was plotted by Nelson-Aalen method. Patients were included just once, at Day0, and time-out was death by any cause or first outcome occurrence. Statistical analysis was performed using the software Stata SE 16.0. P-values < 0.05 were considered statistically significant.

## Results

During the study period, 16,330 cardiac surgeries were performed, and 588 sternal SSI episodes (3.6%) were found. Among them, 14% (84/588) were excluded due to death during index hospitalization and 3% (15/588) due to loss of follow-up (Figure 1). Of the remaining 489, the primary outcome occurred in 14% (67/489) in a median of 174 days (IQR±41.2) after index cardiac surgery. Among them, 73% (49/67) were considered infection relapses, and 27% (18/67) infection persistence, with the last occurring in 15 (IQR±36) days. The mean follow-up was two years (range 0.002 - 489). Demographic, clinical and therapeutic data of index SSI are shown in Table 1 and cumulative incidence in Figure 2.

**Figure 1:** – Study diagram and exclusion criteria.

**Table 1:** – Characteristics of the study population and univariate analysis

Baseline Characteristic/ Variables	All patients n=489 (%)	Therapeutic failure n=67 (13.7%)	No therapeutic failure n=422 (86.2%)	95%CI	p value
**Mean age, years ≥50 years-old**	58.3 (±15) 377 (77.1)	60.8 (±14) 57 (85.0)	57.9 (±15) 10 (14.9)	0.93-3.57	0.14 0.059
**Female**	265 (54.2)	40 (59.7)	225 (53.3)		0.36
**Diabetes mellitus**	185 (37.8)	36 (53.7)	149 (35.3)	1.25-3.28	0.003
**Index cardiac surgery**					
Coronary by-pass	229 (54.0)	40 (69.0)	189 (51.6)	1.11-2.97	0.014
Previous median sternotomy	54 (11.0)	10 (14.9)	44 (10.4)		0.29
**Clinical presentation and treatment of SSI index**					
Incubation period ≥28 days	94 (19.2)	13 (19.4)	81 (19.2)		1.00
Purulent wound drained	427 (88.4)	63 (96.9)	364 (87.1)	1.08-18.1	0.007
Dehiscence ≥ 3cm	52 (10.6)	7 (10.4)	45 (10.7)		1.00
Fever	118 (24.5)	21 (31.7)	97 (23.4)		0.17
Secondary bloodstream infection	89 (19.8)	18 (26.9)	71 (16.)	0.99-2.93	0.06
Mediastinitis/osteomyelitis	88 (18.0)	22 (32.8)	66 (15.6)	1.41-3.93	0.002
Delay of effective antibiotics ≥ 3 days^b^	93 (19.0)	19 (28.4)	64 (17.5)	1.01-2.94	0.05
Debridment surgery	259 (52.9)	42 (62)	217 (51.4)		0.12
**Radiologic findings**					
Sternal misalignment	9 (1.9)	3 (4.5)	6 (1.4)		0.11
Sternal diastasis/dheiscence	34 (7.0)	5 (7.5)	29 (6.9)		0.8
Sternal sclerosis/erosion	9 (1.8)	0	9 (2.1)		0.62
Sternal resorption	9 (1.8)	1 (1.5)	8 (1.9)		1.00
Pre-sternal^c^	131 (26.8)	20 (29.9)	111 (26.4)		0.55
Poststernal^c^	205 (42)	29 (43.3)	176 (41.8)		0.89
**Etiology SSI**^**a**^ **index**					
Positive culture	337 (68.9)	52 (77.6)	285 (67.5)		0.12
S.aureus MRSA	108 (22.1) 18 (3.7)	23 (34.3) 5 (7.5)	85 (20.1) 13 (3.1)	1.14-3.15	0.016 0.085
CoNs	148 (30.3)	16 (23.9)	132 (31.3)		0.25
*Streptococcus* group	11 (2.2)	1 (1.5%)	10 (2.4)		1.00
*GNB GNB* carbapenem-resistant	127 (26) 25 (5.1)	26 (38.8) 8 (11.9)	101 (23.9) 17 (4.0)	1.30-5.72	0.016 0.018
Fungi	23 (4.7)	6 (9.0)	17 (4.0)	0.94-5.06	0.09
Enterococcus species	27 (5.5)	4 (6.0)	23 (5.5)		0.78
**Skin contaminants**	15 (3.1)	3 (4.5)	12 (2.8)		0.44
Lab values at Day 0					
Creatinine (mg/dL)		1.34 (±1.1)	1.32 (±1.32)		0.94
C-reactive protein (mg/L)		123.3 (±90.0)	119.6 (±96.3)		0.79
Leukocyte (mg/L)		11.022 (±4782)	10.826 (±4845)		0.77
Platelets (mg/L)		387.983 (±131)	300.977 (±136)		0.5

CI: confidence interval; OR: Odds ratio; S. aureus: Staphylococcus aureus; MRSA: methicillin-resistant Staphylococcus aureus; CoNs: coagulase-negative Staphylococcus spp.; GNB: Gram-negative bacilli; Skin contaminants: Cutibacterium acnes, Corynebacterium spp., Micrococcus luteus, Propionibacterium sp.; a: Surgical site infection; b: antibiotic adjustments due to bacterial resistance; c: fluid collections and soft tissue swelling.

**Figure 2:** – Nelson-Aalen cumulative incidence curve.

Regarding index sternal SSI, the incubation period was 16 days (± 10) and time to effective antibiotic therapy was six days (±2.8). A total of 259 out of 489 patients (53%) underwent an initial debridement at index SSI. Time from Day0 to first debridement was two days (±1.5), and time to sternal closure was 14 days (±0.7). An average of three surgical procedures were performed per patient. In the subgroup of patients with index sternal SSI classified as mediastinitis/osteomyelitis, 93% (82/88) were debridement at index sternal SSI, with participation of plastic surgeons in 92% (76/82) of the cases; in all of them, vacuum-assisted closure was performed. Among patients with therapeutic failure of index sternal SSI, 62% (42/67) had undergone initial debridement for index SSI treatment. Among the 38% (28/67) who initially had a conservative approach, 42% (12/28) had late debridement due to this previous therapeutic failure. Osteomyelitis occurred in 31% (21/67) of all outcomes and in 95% (21/22) of therapeutic failures in the mediastinitis/osteomyelitis group.

A total of 84% (413/489) index sternal SSIs were cultured; 84.5% (347/413) of them were positive, among which 6.3% (21/347) were considered contamination and excluded. The most common microorganisms were S. aureus (22%), coagulase-negative staphylococci (30%) and gram-negative bacilli (GNB) (26%). Of GNB, *Enterobacteriaceae* were 85%, with *Klebsiella pneumoniae* 41% *and* non-fermenting 21%, with *Pseudomonas aeruginosa* representing 52% of them. Polymicrobial infections (≥ 2 etiologies) occurred in 25% (83/347).

Gram-positive infections were the main attributed cause of therapeutic failure, accounting for 58% (39/67) of outcomes. Among the 39 therapeutic failures, 29 were classified as infection relapse and 10 as infection persistence. *S. aureus* was responsible for 59% (23/39) of therapeutic failures, 70% (16/23) had undergone debridement for treatment of index SSI. GNB occurred in 39% (26/67) of therapeutic failures – 16 were infection relapse and 10 infection persistence. *Enterobacteriaceae* were responsible for 76% (20/26) of outcomes, 70% (14/20) of these due to *Klebsiella pneumoniae.* Among the eight outcomes caused by carbapenem-resistant *Enterobacteriaceae*, 75% (6/8) were *Klebsiella pneumoniae* and 25% (2/8) were *Pseudomonas aeruginosa*. Debridement of index SSI was performed in 100% (8/8) of therapeutics failures in GNB carbapenem-resistant. Fungal etiology occurred in 23 infections and more than 90% (21/23) were due to *Candida spp*., with a prevalence of 66% (14/21) of *C. albicans*. Only one case was due to *Aspergillus fumigatus* and 1 due to *Trichosporon asahii.* All the eight therapeutic failures were due to *C. albicans*, 50% (4/8) of them being considered infection persistence. Initial surgical debridement had been performed in 83% (5/6). The variables that remained statistically significant are presented in Table 2 and in Central Illustration.

**Table 2:** – Multivariable Cox model of factors associated with time to therapeutic failure

Variables	Hazard-ratio	95% Conf. Interval	p value
Diabetes mellitus	2.08	1.28- 3.37	0.003
S. aureus	1.87	1.11-3.17	0.018
Carbapenem-resistant gram-negative bacilli	2.89	1.36-6.12	0.005
Fungi	2.47	1.03- 5.91	0.042
Mediastinitis/osteomyelitis	1.89	1.11-3.21	0.018

## Discussion

The present study suggests an important role of etiology of sternal SSI on treatment failure, mainly if emergent GNB carbapenem-resistant are involved. Additionally, deep sternal wound infection and DM also worsened the infection’s prognosis.

The overall rate of therapeutic failure was 14%; we chose to adopt a combination of outcomes to define therapeutic failure, to focus on the difficulty in treating sternal SSI. Sternal SSI recurrences were discussed in previous studies comparing surgical techniques for management of sternal wounds,^23-26^ frequently without a clear definition. A retrospective cohort study involving 43 cases of post-surgical mediastinitis^25^ showed a rate of 31% of re-infections with a median follow-up of four years. In this study, re-infection was defined as a deep sternal wound infection after at least one adequate treatment attempt or new surgical intervention after hospital discharge. Another retrospective review of 118 poststernotomy mediastinitis^23^ reported a 9% reinfection rate, without specifying the outcome definition. In another study,^24^ 101 patients with post-sternotomy mediastinitis, confirmed by culture, were retrospectively analyzed, with 6% of sternal fistula recurrence. Another cohort study of 92 patients^26^ reported a 9.7% recurrence infection. In this study,^26^ infection recurrence was defined as purulent discharge, positive blood culture in the presence of sternal dehiscence and systemic signs of sepsis. Similarly, one study^20^ with 267 cases of deep sternal wound infections also reported a 9.7% infection recurrence, without a specific infection recurrence definition. Moreover, in our population, the higher proportion of infection relapses with a late occurrence (around six months) is compatible with the high rate of osteomyelitis involved and suggests a scenario of possible subclinical / unsuspected bone invasion regarding the first sternal SSI.

DM is a well-recognized host risk factor for sternal SSI by its impairing wound and bone healing, negatively influencing vascularity and immune function.^12-16^The current study reinforces its role in worsening infection prognosis by showing it as a risk factor for therapeutic failure.

Surgical debridement is nowadays understood as the mainstay therapy for postoperative mediastinitis, with vacuum-assisted therapy showing favorable results regarding lowering infection recurrence.^23,25,26^ One cohort study^23^ of 118 patients reported a significantly reduced re-infection rate among patients treated with vacuum-assisted therapy compared to those treated to conventional therapy, from 18% to 2.9%.^23^ A lowering in re-infection rate from 60% to 10% with vacuum-assisted therapy was seen in a cohort study with 43 patients.^25^ Another study reported lower 90-day mortality when vacuum was used compared to conventional techniques among 101 patients,^24^ but no significant difference in sternal fistula recurrence. In the current case, more than 90% of mediastinitis/osteomyelitis were treated with debridement.

Regarding the microbiology analyses, *S. aureus* was the second most prevalent pathogen involved in sternal SSI, but the most prevalent etiology of therapeutic failure and an independent risk factor for this complication. The NHSN^29^ described *S. aureus* as the most common pathogen for all SSIs, mainly for orthopedic, obstetric/gynecologic, and cardiac surgeries, with an incidence of 27% in the last one. A cohort study reporting 126 cases of post-sternotomy mediastinitis^30^ found a higher proportion of CoNS (46%) and S. aureus as the second main etiology (26%). Simillary,109 sternal wound infections also presented a slightly high prevalence of CoNs (36.7%) compared to *S. aureus* (30%) in another study.^31^ Another cohort study of 291 sternal wound infections^12^ also reported *S. aureus* as the second most common pathogen (16.5%), causing 80% of postsurgical mediastinitis. A systematic review of more than 3500 osteomyelitis^32^ found that *S. aureus* was related to a higher risk of treatment failure in patients with vertebral osteomyelitis. Literature has also drawn attention to mediastinitis caused by methicillin-resistant *Staphylococcus aureus* (MRSA),^33,34^ however, in our study, the role of MRSA in therapeutic failure was not assessed due to its low prevalence.

Postoperative fungal mediastinitis has an incidence ranging from 1.6-7.5% in the few existing reports.^35,36^ A serial case report of 11 patients with deep sternal wound infections due to *C. albicans*^36^ showed a high prevalence of osteomyelitis: six patients had sternal osteomyelitis, one had osteomyelitis and mediastinitis, and four had deep wound infections that probably involved bone. Moreover, three patients experienced infection relapse after six months of antifungal effective therapy. A review of 76 cases of sternal wound infections due to *Candida* spp.^37^ found a 33% primary infection relapse, defined as re-intervention. In the present study, there was a prevalence of 4.7% of *Candida* spp. in sternal SSI. Although its low incidence, it appears as an independent risk factor for therapeutic failure.

Emerging carbapenem-resistant GNBs are generally reported in health-associated infections,^29^ mainly in device-associated infections, with few studies assessing its participation in SSI.^38,39^ A prospective cohort study of 50 patients^38^ assessed carbapenem-resistant *Enterobacteriaceae* infection in abdominal SSI and found a higher mortality rate associated with solid tumor/metastasis, septic shock and blood transfusion. A multicenter national cohort study conducted in Saudi Arabia^39^ showed a 73% prevalence of SSI due to GNB after coronary-bypass, with an incidence of up to 10.8% antimicrobial resistance (including cephalosporin, carbapenem and multidrug resistance patterns). Another cohort study with 33 patients exhibited 33% mortality rate associated with post-surgical mediastinitis caused by carbapenem-resistant GNB.^40^ In a cohort study^41^ with 142 patients with skin and soft tissue infection due to carbapenem resistant *Enterobacteriaceae*, 26 (24.5%) were SSI, which was the second most prevalent wound type. Besides, it showed a 15.5% intra-hospital mortality, and approximately one-half of survivors were discharged to a location to receive higher level supportive care. Our study suggests that, despite its low prevalence, carbapenem-resistant GNB infection is a risk factor for sternal SSI therapeutic failure, as it was detected among patients with high prevalence of surgical debridement, vacuum-assisted closure and prolonged antibiotic therapy. This result is plausible, considering the restricted therapeutic options and more complex clinical conditions associated with this etiology.

### Limitations

Given the retrospective design, the authors were unable to fully account for confounding variables, draw conclusions regarding causality and assess patients’ functional status and clinical progress. As a single center cohort, external validation could not be achieved.

## Conclusion

Emerging carbapenem-resistant GNB, fungi and *S. aureus* were associated with higher risk of therapeutic failure in sternal SSI. In addition, DM and deep sternal wound infections were also contributing factors. Its clinical implications and the exact role of multi-resistant microorganisms should be further investigated.
